# Detecting lncRNA–Cancer Associations by Combining miRNAs, Genes, and Prognosis With Matrix Factorization

**DOI:** 10.3389/fgene.2021.639872

**Published:** 2021-06-28

**Authors:** Huan Yan, Hua Chai, Huiying Zhao

**Affiliations:** ^1^Department of Medical Research Center, Sun Yat-sen Memorial Hospital, Guangzhou, China; ^2^Guangdong Provincial Key Laboratory of Malignant Tumor Epigenetics and Gene Regulation, Guangzhou, China; ^3^School of Data and Computer Science, Sun Yat-sen University, Guangzhou, China

**Keywords:** lncRNA, cancer, prognosis, survival, mutation

## Abstract

**Motivation:** Long non-coding RNAs (lncRNAs) play important roles in cancer development. Prediction of lncRNA–cancer association is necessary for efficiently discovering biomarkers and designing treatment for cancers. Currently, several methods have been developed to predict lncRNA–cancer associations. However, most of them do not consider the relationships between lncRNA with other molecules and with cancer prognosis, which has limited the accuracy of the prediction.

**Method:** Here, we constructed relationship matrices between 1,679 lncRNAs, 2,759 miRNAs, and 16,410 genes and cancer prognosis on three types of cancers (breast, lung, and colorectal cancers) to predict lncRNA–cancer associations. The matrices were iteratively reconstructed by matrix factorization to optimize low-rank size. This method is called detecting lncRNA cancer association (DRACA).

**Results:** Application of this method in the prediction of lncRNAs–breast cancer, lncRNA–lung cancer, and lncRNA–colorectal cancer associations achieved an area under curve (AUC) of 0.810, 0.796, and 0.795, respectively, by 10-fold cross-validations. The performances of DRACA in predicting associations between lncRNAs with three kinds of cancers were at least 6.6, 7.2, and 6.9% better than other methods, respectively. To our knowledge, this is the first method employing cancer prognosis in the prediction of lncRNA–cancer associations. When removing the relationships between cancer prognosis and genes, the AUCs were decreased 7.2, 0.6, and 5% for breast, lung, and colorectal cancers, respectively. Moreover, the predicted lncRNAs were found with greater numbers of somatic mutations than the lncRNAs not predicted as cancer-associated for three types of cancers. DRACA predicted many novel lncRNAs, whose expressions were found to be related to survival rates of patients. The method is available at https://github.com/Yanh35/DRACA.

## Introduction

The human genome consists of protein-encoding mRNA and non-coding RNAs (ncRNAs), but only a small portion of the human genome corresponds to the protein-coding genes (PCGs; Atkinson et al., 2012; Ezkurdia et al., 2014). Among ncRNA, long non-coding RNAs (lncRNAs) are transcription length over 200 nucleotides (Wilusz et al., 2009; Evans et al., 2016) that play important roles in a variety of biological processes and pathological conditions of cancers. The abnormal transcriptions of lncRNA may cause changes in the expression of target genes related to cancer pathways (Prensner and Chinnaiyan, 2011; de Lena et al., 2017). For example, lncRNA *PTENP1* is a pseudogene of the tumor suppressor *PTEN*, which inhibits the induction of autophagy in liver cancers (Chen et al., 2015). Another lncRNA *GAS5* has been shown to regulate cancer proliferation in many human cancer systems (Mazar et al., 2017). In recent years, a portion of lncRNAs has gradually been used as biomarkers of cancers. For example, in human hepatocellular carcinoma cells (HCCs), the lncRNA, uc002mbe.2, is expressed at lower levels than normal cells, but its expression can be increased 300-fold after treatment with histone deacetylase inhibitor Trichostatin A (TSA, Yang et al., 2013). The lncRNA *SChLAP1* is a tissue biomarker that can be used to identify prostate cancer patients at high risk of fatal progression, according to a study of prostate cancer patients in the United States (Mehra et al., 2016). Unfortunately, efficiently identifying lncRNAs–cancers associations is a challenge due to the complexity of relationships between them.

Detecting associations of lncRNAs and common cancers is important for early diagnosis and improving overall survival rate. Currently, breast, lung, and colorectal cancers are the most frequently diagnosed cancers. Although the overall survival rate of breast cancer has improved significantly, it is still an important cause of global death (Kalimutho et al., 2019). Therefore, it is necessary to identify lncRNAs associated with cancers for improving the early diagnosis. In recent years, a growing number of evidences demonstrate that lung cancer is one of the main causes of cancer death in men and women all around the world (Jemal et al., 2011). Simultaneously, colorectal cancer is the third most common cancer worldwide, with 1.36 million people diagnosed in 2012 (Ferlay et al., 2015). Thus, the occurrence of these three types of cancers is a serious threat to human health. Predicting potential lncRNAs associated with these cancers can provide useful information for prevention, diagnosis, and treatment.

Many lncRNAs play important roles through interacting with miRNAs. miRNA is a class of single-stranded RNAs with about 22 long chains of nucleotides, which act as either oncogene or tumor suppressor (Bartel, 2004). Accumulating evidences demonstrated that lncRNA–miRNA crosstalk has emerged as core roles in the pathogenesis and development of human cancer (Xue et al., 2017). Thus, constructing lncRNA–miRNA relationship may help to identify lncRNA–cancer associations.

By using interactions between lncRNA with other molecules, many methods have been developed to predict potential lncRNA–cancer associations (Chen et al., 2017). Liu et al. (2015) proposed a method that utilized the expression profiles of lncRNAs and PCGs in cancers to construct lncRNA–PCG bipartite network, which was then used to identify cancer-associated lncRNAs *via* random walks. It has previously used human phenotypic ontologies to annotate disease to improve the predictive power of lncRNA associated with disease (Le and Dao, 2018). Recently, based on the relationships of lncRNA or miRNA with other molecules, matrix factorization methods were used to predict lncRNA–disease associations (Fu et al., 2018) and miRNA–disease associations (Xuan et al., 2019). LION model applied the characteristics of lncRNAs, genes, and diseases to predict the relationships between lncRNAs and diseases through network diffusion (Sumathipala et al., 2019). At the same time, there are also related study based on heterogeneous clustering methods to predict the unknown relationships between lncRNAs and diseases based on the relationship network constructed by diseases, lncRNAs, microRNAs, and genes (Barracchia et al., 2018). LP-HCLUS uses multi-type hierarchical clustering methods to predict potentially lncRNA–disease relationships (Barracchia et al., 2020). However, all these methods only discriminate disease-associated lncRNAs without relating the lncRNAs with specific cancer types.

Moreover, all these methods overlooked the relationships between lncRNAs and cancer prognosis. The presence of lncRNAs in cancers can be an important factor clinically determining the prognosis of patients. Recently, an approach has been proposed to estimate the relationship between genes and the cancer prognosis by analyzing multi-omics data and clinical information from The Cancer Genome Atlas (TCGA) database (Wang et al., 2018). More recently, a method was presented to determine the gene and patient prognosis for 13 types of cancers (Chai et al., 2019), which reminds us to use the relationships between genes and the prognosis of three types of cancers in the prediction of lncRNA–cancer association.

In this study, we constructed a method, called detecting lncRNA cancer association (DRACA), to predict associations between lncRNAs and three common cancers. This method integrated the relationships between lncRNAs, cancer prognosis, miRNAs, genes, and cancers into a matrix and utilized matrix factorization to fuse multiple effective biological features in the prediction. This is the first method using cancer prognosis to detect lncRNA–cancer associations, which was indicated as a critical feature in the prediction. Further analyses indicated that the predicted cancer-associated lncRNAs contain significantly more somatic mutations than the average. In addition, several novel cancer-associated lncRNAs predicted by this study were significantly correlated with the survival rates of cancer patients and were expressed to be significantly different in cancer tissues and paracarcinomatous tissues. Thus, the predicted lncRNAs are biologically meaningful in the cancer process.

## Methods

### Matrix Factorization

The matrices were constructed by the relationships between *N* (*N* = 5) kinds of features. The main framework of the model is to optimize the equation:

(1) $${\overset{min}{G}}_{\geq 0}\text{O(}G\text{, }S\text{, }W\text{)}=\sum_{R_{ij}\in ℛ}W_{ij}{||R_{ij}-G_{i}S_{ij}G_{j}^{T}||}_{F}^{2}+\alpha {||vec\left(W\right)||}_{F}^{2}$$

s.t.W≥0,∑vec(W)=1

where α is used to control the complexity of *vec*(*w*) (set as 1×10^5^ in the study), *R*_*ij*_ is a collection of relations across data sources that include *R*_*LM*_, *R*_*LG*_, *R*_*LC*_, *R*_*GP*_, *R*_*MG*_, *R*_*MC*_, and *R*_*GC*_ (Table 1), *i* and *j* are the *i*th and *j*th features from two different data sources, respectively, *R*_*ij*_ is reconstructed as $G_{i}S_{ij}G_{j}^{T}$ by singular vector decomposing (SVD), *W* is calculated by Equation 2, *i* and *j* are two kinds of features, and $||\cdot |{|}_{F}^{2}$ is the Frobenius norm.

**TABLE 1:** The matrix size and the number of associations in the dataset.

**Relationships**	**Matrices**	**Size**	**Associations**
lncRNA–miRNA	*R*_*LM*_	1,679 × 2,759	10,120
lncRNA–gene	*R*_*LG*_	1,679 × 16,410	511
lncRNA–cancer	*R*_*LC*_	1,679 × 3	542
miRNA–gene	*R*_*MG*_	2,759 × 16,410	380,639
miRNA–cancer	*R*_*MC*_	2,759 × 3	3,343
Gene–cancer	*R*_*GC*_	16,410 × 3	9,015
Gene–prognosis	*R*_*GP*_	16,410 × 3	1,169

The low-rank size of reconstructed matrix in Equation 1 was optimized according to the prediction of lncRNA–cancer relationships in the training set by giving appropriate weights (*W*_*ij*_). *W*_*ij*_ was calculated by Equation 2, where γ is the Lagrangian multipliers. Here, the performance of the prediction was evaluated by Area Under Curve (AUC). To avoid overfitting, 10-fold cross-validation was employed.

(2) $$w_{ij}=\left\{ \begin{array}{c} \frac{\gamma -H_{ij}}{2\alpha },if\gamma -H_{ij}>0andR_{ij}\in R\\ 0,if\gamma -H_{ij}\leq 0andR_{ij}\notin R \end{array}\right.$$

$$\left(H_{ij}={||R_{ij}-G_{i}S_{ij}G_{j}^{T}||}_{F}^{2}\right)$$

### Dataset Construction

The dataset includes five kinds of features and their relationships, which are lncRNAs, miRNAs, genes, cancers, and cancer prognosis. The relationships between these features were collected from public databases. The lncRNA–miRNA relationships (*R*_*LM*_) were downloaded from starBase v2.0 (Li et al., 2014); the lncRNA–gene interactions (*R*_*LG*_) were from lncReg (Zhou et al., 2015); the lncRNA–cancer associations (*R*_*LG*_) were from lncRNADisease (Bao et al., 2018); the miRNA–gene relationships (*R*_*MG*_) were from miRTarbase (Chou et al., 2018); the miRNA–cancer relationships (*R*_*MC*_) were from MNDR v2.0 (Cui et al., 2018); the gene–cancer (*R*_*GC*_) relationships were from DisGeNet (Pinero et al., 2017).

Additionally, we calculated the gene–prognosis relationships (*R*_*GP*_) by integrating multi-omics data from TCGA as described in a previous study (Chai et al., 2019). Briefly, we downloaded multi-omics data including RNA expression data, DNA methylation data, and copy number variation data of 614 breast cancer patients, 733 lung cancer patients, and 255 colorectal cancer patients from TCGA dataset^1^; then, we employed Autoencoder to rebuild composite features that were subsequently used by Cox proportional hazard model to estimate the prognosis risk of patients. Finally, XGboost was used to classify the prognosis of patients into high and low risks by scoring relationships between genes and the prognosis. The scores of genes were ranged from 0 to 1. The genes with scores higher than 0.5 were defined as highly correlated. The relationships between the genes and the prognosis of three kinds of cancers were included in the matrix factorization model. In summary, this study constructed a dataset including 1,679 lncRNAs, 2,759 miRNAs, 16,410 genes, and 16,410 genes–prognosis relationships and three kinds of cancers (breast, lung, and colorectal).

**FIGURE 1:**
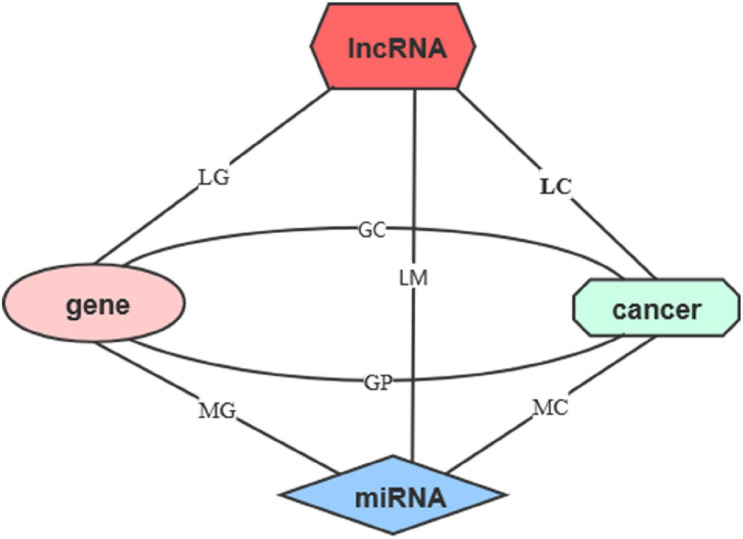
The network of five features. The five features include lncRNAs, miRNAs, genes, cancers, and cancer prognosis. The line represents the relationship matrices.

The relationships between these data are provided in Table 1. By using these relationships, we constructed lncRNA–cancer network as shown in Figure 1. The lncRNA–cancer relationships in lncRNADisease were used as golden standards to determine the lncRNA–cancer associations. As shown in Table 1, **542** lncRNA–cancer associations in the database were considered as the positive dataset, and 4,495 lncRNA–cancer with no relationships were included as the negative dataset. Briefly, 185, 179, and 178 lncRNAs associated with breast cancer, lung cancer, or colorectal cancer were collected as the positive dataset, whereas 1,494, 1,500, and 1,501 lncRNAs not associated with breast cancer, lung cancer, or colorectal cancer were collected as the negative dataset.

### Statistical Measurements in Evaluating the Methods

The 10-fold cross-validation was used to evaluate the performance of DRACA. We randomly divided positive and negative genes into 10-fold and used nine-fold as training and one-fold for testing. This process was repeated for 10 times. The prediction AUC was calculated for the testing fold. The average AUC was used as 10-fold cross-validation result of the model. In this study, we used AUC, maximum Matthews correlation coefficient (MCC), accuracy (ACC), precision, sensitivity, and specificity to evaluate the performance of DRACA. Calculations of these measurements were shown in Equations 3–7.

(3) $$MCC=\frac{TP\times TN-FP\times FN}{\sqrt{\left(TP+FP\right)\left(TP+FN\right)\left(TN+FP\right)\left(TN+FN\right)}}$$

(4) $$ACC=\frac{TP+TN}{TP+TN+FP+FN}$$

(5) $$precision=\frac{TP}{TP+FP}$$

(6) $$sensitivity=\frac{TP}{TP+FN}$$

(7) $$specificity=\frac{TN}{FP+TN}$$

## Results

### The Influences of the Low-Rank Size (k)

The low-rank size (*k*) of decomposed matrix in Equation 1 was optimized according to the performance of prediction. The performance was evaluated by AUC. In this study, *k*_1_ was the low-rank size of *R*_*[lncRNA]*_ that was the relationship between lncRNA with other features and was kept as 1,679; *k*_4_ and *k*_5_ were the low-rank sizes of *R*_*[cancer]*_ and *R*_*[cancer  prognosis]*_ that were the relationships between cancers with other features and were kept as 3. *k*_2_ and *k*_3_ were the low-rank sizes of *R*_*[miRNA]*_ and *R*_*[gene]*_ that were relationships between miRNA and gene with other molecules and cancers, respectively. *k*_2_ and *k*_3_ were optimized.

The *k*_2_ was optimized from 10 to 2,759 by a step of 100 and keeping *k*_3_ as 50 to reduce the computational cost. As a result, when *k*_2_ = 1,610, the highest AUC of 0.787 was achieved. Then, *k*_3_ was trained by keeping *k*_2_ = 1,610. The best AUC of 0.789 was provided when *k*_3_ = 1,810. Then, we examined the performance of the model in predicting the lncRNA associations with breast cancer, lung cancer, and colorectal cancer, respectively. AUC values of 0.806, 0.801, and 0.778 were achieved, respectively, for three types of cancers.

We expected that the model gave a better performance when it was trained for a specific cancer. Here, this model was trained for prediction of associations between lncRNA and breast cancer, lncRNA and lung cancer, and lncRNA and colorectal cancer, respectively. In the training procedure, *k*_2_ and *k*_3_ were optimized, and 10-fold cross-validation was applied to avoid over training. For breast cancer, when *k*_2_ = 2,210 and *k*_3_ = 2,510, the highest AUC of 0.810 was obtained, which was slightly higher than the AUC of 0.806 obtained by the model trained for predicting all associations between the cancers and lncRNA. For lung cancer, when *k*_2_ = 1,110 and *k*_3_ = 3,110, the AUC was 0.796 that was a marginal decrease compared with 0.801 obtained by the model trained for prediction of all associations between the cancers and lncRNA. For colorectal cancer, *k*_2_ = 1,610 and *k*_3_ = 710 provided the highest AUC of 0.795 that was higher than the AUC of 0.778 reached by predicting all associations between the cancers and lncRNA. The results are shown in Table 2. We further used this method in liver hepatocellular carcinoma. Result indicated that the 10-fold cross-validation AUC achieved 0.749 and MCC achieved 0.313 (Table 2).

**TABLE 2:** The performance of DRACA in the prediction of associations between lncRNA and three types of cancers.

**Cancer**	**AUC (AUC^*a*^)**	**MCC**	**ACC**	**Precision**	**Sensitivity**	**Specificity**
Breast cancer	0.810 (0.806)	0.336	0.658	0.232	0.910	0.625
Lung cancer	0.796 (0.801)	0.404	0.764	0.294	0.858	0.764
Colorectal cancer	0.795 (0.778)	0.371	0.714	0.254	0.888	0.694
Liver hepatocellular carcinoma	0.749	0.313	0.676	0.236	0.841	0.656

*^*a*^The AUC values of the DRACA model that was trained to predict the association between lncRNA and three cancers.*

### Measuring the Contribution of the Features

To measure the contribution of each feature in the prediction, we individually removed the relationships between features and examined their influence on AUC areas. For prediction of breast cancer-associated lncRNAs, when the relationship between genes and cancer prognosis (*R*_*GP*_) was removed, the AUC of DRACA was reduced from 0.810 to 0.738 (7.20%). In removing the relationship *R*_*GP*_ in the prediction of lung cancer, the AUC was reduced from 0.796 to 0.790 (0.60%). In the prediction of lncRNA–colorectal cancer association, the removal of *R*_*GP*_ dramatically reduced the AUC values from 0.795 to 0.745 (5.00%). We also examined the contributions of the relationships, *R*_*LM*_, *R*_*LG*_, and *R*_*MG*_, in the prediction of the associations of lncRNA with three types of cancers, respectively. The results are shown in Table 3. As shown in Table 3, the lncRNA–miRNA (*R*_*LM*_) was the most important feature in the prediction. Meanwhile, we found that removing the gene–cancer relationships or miRNA–cancer relationships can also reduce the prediction.

**TABLE 3:** The AUCs and MCCs for DRACA predictions after removing the associations between features.

	**Breast cancer**		**Lung cancer**		**Colorectal cancer**	
	**AUC**	**MCC**	**AUC**	**MCC**	**AUC**	**MCC**
All	0.81	0.336	0.796	0.404	0.795	0.371
-*R*_*LM*_	0.57	0.048	0.585	0.056	0.549	−0.010
-*R*_*LG*_	0.749	0.333	0.756	0.356	0.731	0.312
-*R*_*MG*_	0.668	0.258	0.685	0.313	0.569	0.154
-*R*_*GP*_	0.738	0.347	0.79	0.387	0.745	0.303
-*R*_*MC*_	0.715	0.338	0.734	0.339	0.722	0.294
-*R*_*GC*_	0.5	0	0.5	0	0.5	0

When all the miRNA-related features (lncRNA–miRNA, miRNA–gene, and miRNA–cancer features) were removed from the prediction or all the gene-related features (gene–cancer, gene–prognosis, gene–cancer, and miRNA–gene features) were removed from the prediction, the AUC values of DRACA are close to random. More details are included in Supplementary Table 1.

### The Impact of Other Cancers on the Prediction

**FIGURE 2:**
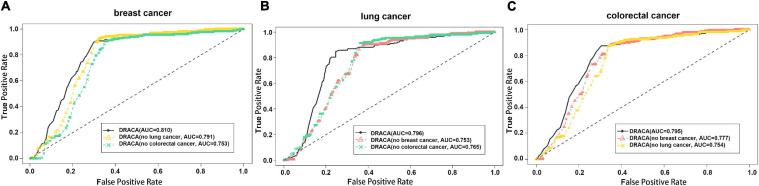
The influences of cancer types in detecting lncRNA cancer association (DRACA) prediction. The black lines represent the ROC curves of DRACA without removing any features in predicting associations between lncRNA and cancers. The red and green lines denote the ROC curves of DRACA removing cancer information. **(A)** The ROC curves to show the influences of removing lung cancer information (yellow curve) or removing colorectal cancer information (green curve) in predicting breast cancer associated lncRNAs; **(B)** The ROC curves to show the influences of removing breast cancer (red curve) or removing colorectal cancer information (green curve) in predicting lung cancer associated lncRNAs; **(C)** The ROC curves to show the influences of removing breast cancer information (red curve) or lung cancer (yellow curve) in predicting colorectal cancer associated lncRNAs.

This study constructed DRACA by including the information of three types of cancers that may have influences on the prediction. These influences were tested through excluding cancer information individually. As shown in Figure 2, in the prediction of lncRNA–breast cancer associations, removing the lung cancer and removing the colorectal cancer individually resulted in the AUCs of 0.791 and 0.753, respectively, which are lower than the AUC value 0.810 obtained by using all the features. Figure 2 also describes the impacts of breast cancer and colorectal cancer in the prediction of lung cancer-associated lncRNA and the impacts of breast cancer and lung cancer in the prediction of colorectal cancer-associated lncRNAs. When removing breast cancer or colorectal cancer information in predicting lung cancer-associated lncRNAs, the AUC values were decreased from 0.796 to 0.753 or from 0.796 to 0.765, respectively. The contributions of breast cancer and lung cancer in the prediction of lncRNAs associated with colorectal cancer were indicated by the reduced AUCs from 0.795 to 0.777 and to 0.754, respectively. Thus, colorectal cancer contributed more in the predictions of lncRNA–breast cancer and lncRNA–lung cancer associations than two other cancers. Moreover, removing lung cancer had reduced more AUC values in predicting lncRNA–colorectal cancer associations than in removing breast cancer.

### Comparison With Other Methods

Detecting lncRNA cancer association was compared with the Naïve Bayesian classifier to predict potential lncRNA–disease associations (NBCLDA; Yu et al., 2018) in terms of MCC on the same dataset by 10-fold cross-validation. NBCLDA is a method constructing a global tripartite network that combines lncRNA–cancer, miRNA–cancer, and miRNA–lncRNA associations, including gene–miRNA interactions, gene–lncRNA associations, and gene–disease interactions, to predict potential lncRNA–disease associations. Table 4 uncovers that DRACA always performed better in MCCs (0.336, 0.404, and 0.371) than NBCLDA (0.265, 0.256, and 0.245).

**TABLE 4:** Comparing DRACA with three methods on MCC values.

	**Breast cancer**	**Lung cancer**	**Colorectal cancer**
DRACA	0.336	0.404	0.371
NBCLDA	0.265	0.256	0.245
BPLLDA	0.330	0.248	0.393
MFLDA	0.161	0.141	0.057

We also compared the predictions of DRACA with the method developed by integratinglncRNA—disease network, lncRNA functional similarity network, and the disease semantic similarity network (BPLLDA, Xiao et al., 2018). This method inferred the lncRNA–disease association according to the paths connecting them and their lengths in the network. BPLLDA was developed based on a database including 156 lncRNAs and their associated diseases. Among these lncRNAs, 56 were included in the DRACA database, which were used to compare these two methods. The comparison was performed by 10-fold cross-validation and measured by MCC. As shown by Table 4, DRACA performed significantly better than BPLLDA in the prediction of lncRNA–breast cancer associations, lncRNA–lung cancer associations, and lncRNA–colorectal cancer associations.

Furthermore, we compared DRACA with the method developed to predict the lncRNA-disease associations based on matrix factorization approaches MFLDA (Fu et al., 2018). It is different from DRACA in two respects. First, it is a method without considering the relationship between lncRNA and cancer prognosis. Second, it has been constructed by 214 lncRNAs that is much less than the number of lncRNAs in DRACA. Out of 214 lncRNAs, 98 were from the DRACA database, which were used for the comparison. The results indicated that DRACA was superior to MFLDA in predicting the relationships between lncRNAs and three types of cancers.

In summary, DRACA was compared with three recently developed methods in predicting lncRNA–cancer associations. The results indicated that DRACA performed always better than NBCLDA, BPLLDA, and MFLDA in the prediction of three types of cancers. Moreover, DRACA has been constructed by 1,679 lncRNAs that are 7 and 11 times more than lncRNAs in BPLLDA and MFLDA, respectively. Thus, DRACA can potentially discover more novel lncRNA–cancer associations.

### Testing the Predicted lncRNA–Cancer Associations

Detecting lncRNA cancer association gives each lncRNA a score to indicate its relationship with certain cancer. The higher the score, the higher the probability that the lncRNA and the cancer are related. In order to select candidate lncRNAs, we used the maximum MCC to obtain the score threshold. The MCC was calculated by Equation 3. The best MCCs of 0.336, 0.404, and 0.371 were achieved for breast cancer, lung cancer, and colorectal cancer, respectively. When DRACA achieved the best MCC, we also calculated other statistical measurements including accuracy (ACC), precision, sensitivity, and specificity, as shown in Table 2.

By using the thresholds given by the best MCCs for the three types of cancers (0.785, 0.965, and 0.815), 636, 521, and 616 lncRNAs were predicted as related to breast cancer, lung cancer, and colorectal cancer, respectively. From them, we checked the top 20 candidate lncRNAs (a total of 60 lncRNAs for three types of cancers) that were not collected in the lncRNADisease database. We searched these lncRNAs in PubMed to obtain the literatures regarding their relationships with cancers. For breast cancer, lung cancer, and colorectal cancer, respectively, 10, 10, and 13 out 20 lncRNAs were reported as related with cancers. More details are included in Supplementary Tables 2–4.

**FIGURE 3:**
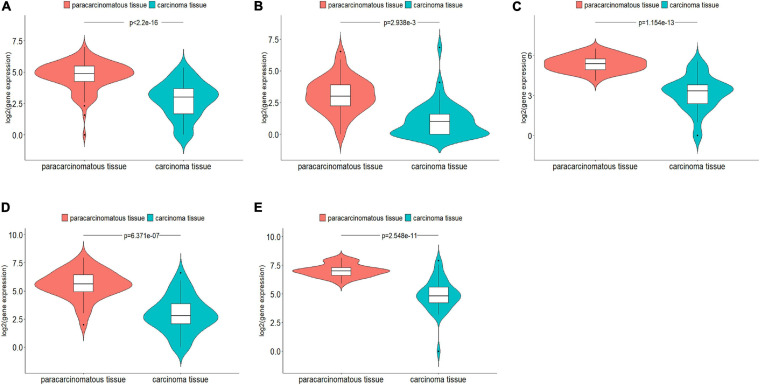
Five genes that were predicted as cancer-associated by DRACA were found expressed significantly different between carcinoma tissues and paracarcinomatous tissues. **(A)** *Lnc-LAMC2-1:1* was found expressed significantly different in breast cancer tissues and paracarcinomatous tissues; **(B)** *DGKK* expressed significantly different in lung cancer tissues and paracarcinomatous tissues; **(C–E)** *EPB41L4A-AS2*, *MANCR*, and *lnc-HOXC4-3:1* expressed significantly different in colorectal cancer tissues and paracarcinomatous tissues.

For these predicted new lncRNAs, we examined if they were expressed to be significantly different in carcinoma tissues and paracarcinomatous tissues. Out of 60 predicted top cancer-associated lncRNAs, 20 were included in TCGA database, which included seven predicted as associated with breast cancer, five predicted as associated with lung cancer, and eight predicted as associated with colorectal cancer. From TCGA database, we downloaded gene expression data for 106 breast cancer patients, 52 lung cancer patients, and 38 colorectal patients. By comparing the gene expression data of these 20 lncRNAs in the carcinoma tissues and the paracarcinomatous tissues using *edgeR R* package (*FDR* < 0.05, | log*FC*| > 1), five lncRNAs were found to be expressed significantly different, which included one lncRNA for breast cancer, one lncRNA for lung cancer, and three lncRNAs for colorectal cancer (Figure 3). The statistical evaluations on the differences of gene expression are shown in Supplementary Table 5.

**FIGURE 4:**
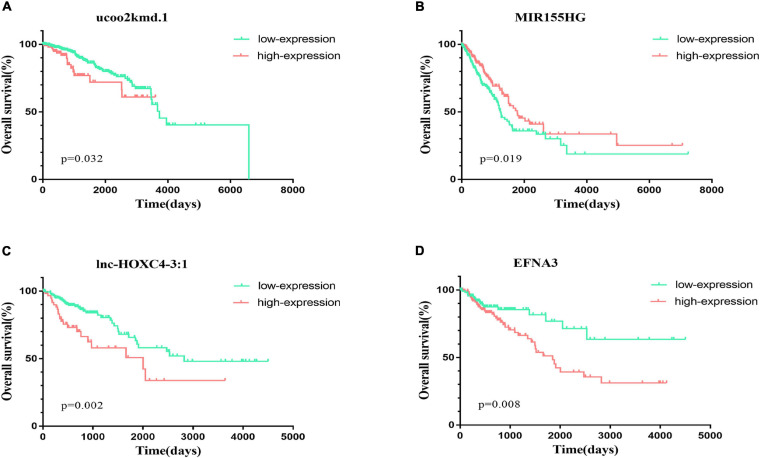
The survival curves of two groups of patients who highly and lowly expressed *ucoo2kmd.1*, *MIR155HG*, *lnc-HOXC4-3:1*, and *EFNA3*, respectively. The patients were divided into two groups using the *surv_cutpoint* function of the *survminer R* package according to the gene expression levels, which were represented as “High-expression” and “Low-expression,” respectively. The red lines denote the relationship between survival time and overall survival for the patients in the “High-expression” group, and the blue lines represent the relationship between the survival time and the overall survival for the patients in the “Low-expression” group. **(A)** The survival curves of two groups of the breast cancer patients who highly and lowly expressed *ucoo2kmd.1* gene, respectively; **(B)** The survival curves of two groups of the lung cancer patients who highly and lowly expressed *MIR155HG* gene, respectively; **(C)** The survival curves of two groups of the colorectal cancer patients who highly and lowly expressed *lnc-HOXC4-3:1* gene, respectively. **(D)** The survival curves of two groups of the colorectal cancer patients who highly and lowly expressed *EFNA3* gene, respectively.

We also analyzed the relationships between 20 lncRNAs and the patient survival rates. From TCGA database, we downloaded survival information for 611 breast cancer patients, 439 lung cancer patients, and 251 colorectal cancer patients. Patients were divided into the high-expression group and low-expression group by using the *surv_cutpoint* function of the *survminer R* package according to the gene expression. Then, we compared the overall survival rates of two groups. The results were shown in Kaplan–Meier plots (Figure 4). The differences of the survival rates were tested by the log-rank (Mantel–Cox) test. Here, the overall survival rates were the numbers of cases living for a certain period divided by the total numbers of patients in this group at the beginning. Genes were defined as significantly related with patient survival rates if the Mantel–Cox test *P*-value is lower than 0. Out of 20 genes, 5 were found to be significantly related with the patient survival rates. Briefly, patients in the low-expression and high-expression groups of *ucoo2kmd.1* were found to be significantly different in survival rates according to Mantel–Cox test (*P*-value = 0.032) as shown in Figure 4A. Similarly, the expression of *MIR155HG* (Figure 4B) was found to be significantly (*P*-value = 0.019) associated with the overall survival of lung cancer. At the same time, the expressions of *lnc-HOXC4-3:1* (Figure 4C), *EFNA3* (Figure 4D), and *LINC00520* (Supplementary Figure 6) were identified to be significantly related with the overall survival of colorectal cancer patients with *P*-values of 0.002, 0.008, and 0.021, respectively. Among these genes, *lnc-HOXC4-3:1* and *EFNA3* were also found to be expressed significantly different in carcinoma tissues and paracarcinomatous tissues as shown in Figure 3C.

### The Numbers of Somatic Mutations in lncRNAs Predicted as Cancer-Associated by Detecting lncRNA Cancer Association

**FIGURE 5:**
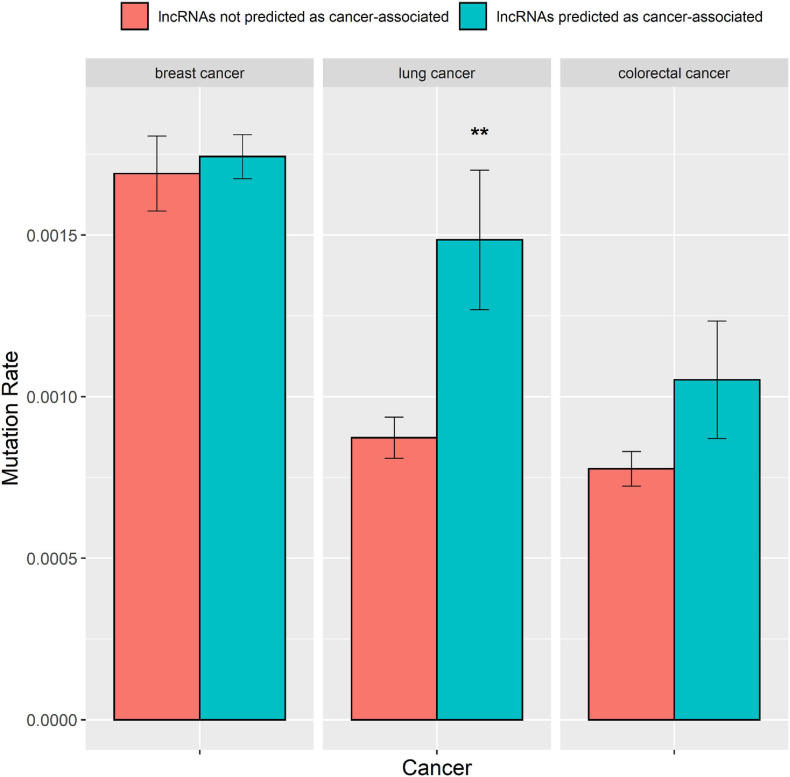
The mutation rates in the lncRNAs predicted as cancer-associated by DRACA are higher than in the lncRNAs not predicted as cancer-associated. “*” denotes *t*-test *P*-value < 5.0–2E; “**” represents *t*-test *P*-value < 1.0–2E.

A greater number of mutations in lncRNAs raise their probability for causing cancers (Beroukhim et al., 2010; Huarte, 2015). Hence, we explored whether the predictions of the DRACA model are correlated with the number of mutations in lncRNAs. We collected somatic mutation data from the international cancer genome consortium (ICGC) database, which contained somatic mutations of 651 lncRNAs for breast cancer, 568 lncRNAs for lung cancer, and 526 lncRNAs for colorectal cancer. Then, we examined the difference between the number of mutations in the lncRNAs that were predicted as cancer-associated and in the lncRNAs that were not predicated as cancer-associated by DRACA. The lncRNAs were defined as cancer-associated if their scores were higher than the threshold giving the best MCC. For three types of cancers, the numbers of mutations in the lncRNAs that are predicted as cancer-associated are higher than those in the lncRNAs that are not predicted as cancer-associated. The lncRNAs predicted as breast cancer-, lung cancer-, and colorectal cancer-associated were indicated with more somatic mutations than the lncRNAs not predicted as cancer related with *P*-values, 3.5e-1, 3.5e-3, and 7.4e-2 (Figure 5). Thus, the lncRNAs predicted as cancer-associated tend to occur with more somatic mutations.

## Conclusion

In this study, we presented a method, DRACA, that is an approach using miRNAs, genes, lncRNAs, and cancer prognosis to construct matrices in the prediction of lncRNA–cancer associations. DRACA utilizes matrix factorization technology to decompose different heterogeneous data matrices into low-rank matrices by tri-factorization and optimizing weight for matrices. Using 10-fold cross-validation, we searched the appropriate sizes of low-rank matrices and verified the validity of the features. In a 10-fold cross-validation experiment, the method obtains AUCs of 0.810, 0.796, and 0.795 in predicting lncRNA-related breast cancer, lung cancer, and colorectal cancer. DRACA was compared with three methods, NBCLDA, BPLLDA, and MFLDA, and was indicated with significantly better performances. To illustrate the biological meaning of the prediction, we compared the predicted score with the number of somatic mutations in each lncRNA. We found that the lncRNAs predicted as cancer-associated have more somatic mutations than the lncRNAs not predicted as cancer-associated. Thus, integrating the relationships among lncRNAs, miRNAs, genes, and cancer prognosis with matrix factorization technology can accurately predict potential lncRNA–cancer associations. Moreover, among 20 novel lncRNAs predicted as cancer-associated by DRACA, nine were indicated to be expressed significantly different between the carcinoma tissues and the paracarcinomatous tissues, and five were significantly correlated with the survival rates of patients.

## Discussion

lncRNAs had been viewed as “junk” in the genome. Recently, lncRNAs have attracted much attention due to the discovery that they are key regulators of cancer transformation and progression. Thus, discovering novel lncRNA–cancer association has possibilities to lead to early diagnosis and new treatment of cancers. Despite the rapid increase in the catalog of roles reported for lncRNAs, one of the greatest challenges is in the identification of cancer risk lncRNAs efficiently.

In this study, we presented an approach, DRACA, to predict lncRNAs associated with three specific cancers. DRACA is different from previously developed methods in several aspects. DRACA includes the feature of cancer prognosis, which greatly improves prediction ability but was missed by other methods. We used AUC to train the model and calculated the best MCC for each model. AUC and MCC are commonly used for evaluating the reliability of the model (Chicco and Jurman, 2020). However, MCC is easy to be fluctuated because MCC value is dependent on the prediction of score of each gene.

## Data Availability Statement

The original contributions presented in the study are included in the article/Supplementary Material, further inquiries can be directed to the corresponding author.

## Author Contributions

HZ designed and supervised the study. HY and HC conducted the analyses. HY wrote the manuscript. All authors contributed to the final revision of the manuscript.

## Conflict of Interest

The authors declare that the research was conducted in the absence of any commercial or financial relationships that could be construed as a potential conflict of interest.

## Footnotes

1 https://www.cancer.gov/tcga
