# Supplementary material for: Detecting lncRNA–Cancer Associations by Combining miRNAs, Genes, and Prognosis With Matrix Factorization
Source: Front Genet. 2021 Jun 28;12:639872. doi: 10.3389/fgene.2021.639872 (PMC8273282; doi:10.3389/fgene.2021.639872)
Supplement: Supplementary file 1 [file Data_Sheet_1.docx]

**Supplementary Table S1** AUC values for DRACA predictions after removing different features

| multi-feature | breast_cancer  AUC | lung_cancer  AUC | colorectal_cancer  AUC |
| --- | --- | --- | --- |
| All | 0.810 | 0.796 | 0.795 |
| -prognosis | 0.738 | 0.790 | 0.745 |
| -miRNA | 0.605 | 0.584 | 0.527 |
| -gene | 0.500 | 0.500 | 0.500 |

**Supplementary Table S2** The top 20 candidate lncRNAs of breast cancer

| LncRNA | Related cancers | References |
| --- | --- | --- |
| AP003774.1 | Acute myeloid leukemia | (Wang et al., 2018a) |
| RP11-133N21.10 |  |  |
| Z83851.3 |  |  |
| RP11-214K3.21 |  |  |
| AC068491.1 |  |  |
| LINC00846 |  |  |
| RP11-473M20.16 | Lung adenocarcinoma | (Xu et al., 2014) |
| RP11-693J15.5 |  |  |
| CTD-2017D11.1 |  |  |
| RP11-21J18.1 |  |  |
| RP6-65G23.3 |  |  |
| lncRNA_ES3 | Breast cancer | (Keshavarz et al., 2019) |
| AC069513.3 | Clear cell renal cell carcinoma | (Shi et al., 2017) |
| LINC00858 | Lung cancer | (Xue et al., 2019) |
| LncRNA-BCAT1 | Colorectal cancer | (Xie et al., 2017) |
| ucoo2kmd.1 | Colorectal cancer | (Wu et al., 2016) |
| LINC01296 | Bladder cancer | (Wang et al., 2019) |
| POU5F1P4 | Breast cancer | (Saha et al., 2018) |
| RP11-785D18.3  lnc-LAMC2-1:1 | Colorectal cancer | (Gong et al., 2016) |

**Supplementary Table S3** The top 20 candidate lncRNAs of lung cancer

| LncRNA | Related cancers | References |
| --- | --- | --- |
| RP11-613D13.8 | Gastric cancer | (Sun et al., 2017) |
| RP11-301L7.1 |  |  |
| RP11-408B11.2 | Lung adenocarcinoma | (Shi et al., 2018) |
| MIR155HG | Glioma | (Wu et al., 2017) |
| RP11-18F14.2 |  |  |
| AC009501.4 |  |  |
| DGKK |  |  |
| AC007566.10 |  |  |
| XLOC_000303 | Colorectal cancer | (Shi et al., 2015) |
| RP11-481J13.1 |  |  |
| uc.388 |  |  |
| CRNDE-h | Colorectal cancer | (Liu et al., 2016) |
| GAPLINC | Colorectal cancer  Gastric cancer | (Luo et al., 2018b)  (Hu et al., 2014) |
| RP11-317J10.2 | Colorectal cancer | (Luo et al., 2018a) |
| LINC00485 | Lung adenocarcinoma | (Zuo et al., 2019) |
| AC079922.3 |  |  |
| CTC-550B14.6 |  |  |
| RP11-307E17.8 |  |  |
| CTC-524C5.2 |  |  |
| EMX2OS | Papillary thyroid cancer | (Gu et al., 2018) |

**Supplementary Table S4** The top 20 candidate lncRNAs of colorectal cancer

| LncRNA | Related cancers | References |
| --- | --- | --- |
| RP11-79N23.1 |  |  |
| lncRNA_ES3 | Breast cancer | (Keshavarz et al., 2019) |
| AC015849.16 |  |  |
| lnc-HOXC4-3:1 | Breast cancer | (Zhong et al., 2016) |
| PCAN-1 | Prostate cancer | (Cross et al., 2004) |
| MANCR | Breast cancer | (Tracy et al., 2018) |
| PCAN-4 |  |  |
| EPB41L4A-AS2 | Breast cancer | (Xu et al., 2016) |
| LSINCT2 |  |  |
| EFNA3 | Breast cancer | (Gomez-Maldonado et al., 2015) |
| lincRNA-BC2 | Breast cancer | (Ding et al., 2014) |
| LINC00520 | Nasopharyngeal carcinoma  Renal cell carcinoma | (Xie et al., 2019)  (Chen et al., 2018) |
| STXBP5-AS1 | Cervical cancer  Non-small-cell lung carcinoma | (Shao et al., 2019)  (Huang et al., 2018) |
| KCNK15-AS1 | Pancreatic Cancer | (He et al., 2018) |
| RP11-351I21-11 |  |  |
| lncRNA-CTD-2108O9.1 | Breast cancer | (Wang et al., 2018b) |
| uc.346+ |  |  |
| SRA1 | Cervical squamous cell carcinoma | (Liu et al., 2019) |
| MSR1 | Breast and prostate cancer | (Rose et al., 2018) |
| AC104135.3 | Breast cancer | (Jiang et al., 2017) |

S**upplementary Table S5** The statistical evaluations on the differences of gene expressions in carcinoma tissues and paracarcinomatous tissues.

| cancer | gene | logFC | logCPM | PValue | FDR |
| --- | --- | --- | --- | --- | --- |
| Breast cancer | lnc-LAMC2-1:1 | 1.731586102 | -1.36431073 | 6.58593E-29 | 7.79891E-28 |
| Lung cancer | DGKK | 1.851305904 | -1.96635845 | 1.51737E-06 | 4.50948E-06 |
| Colorectal cancer | MANCR | 1.742222831 | -0.10020014 | 1.50E-07 | 5.53E-07 |
| Colorectal cancer | EPB41L4A-AS2 | 1.542716097 | -0.58805103 | 7.70E-17 | 9.20E-16 |
| Colorectal cancer | lnc-HOXC4-3:1 | 1.448190275 | 1.07598128 | 2.10E-14 | 1.89E-13 |


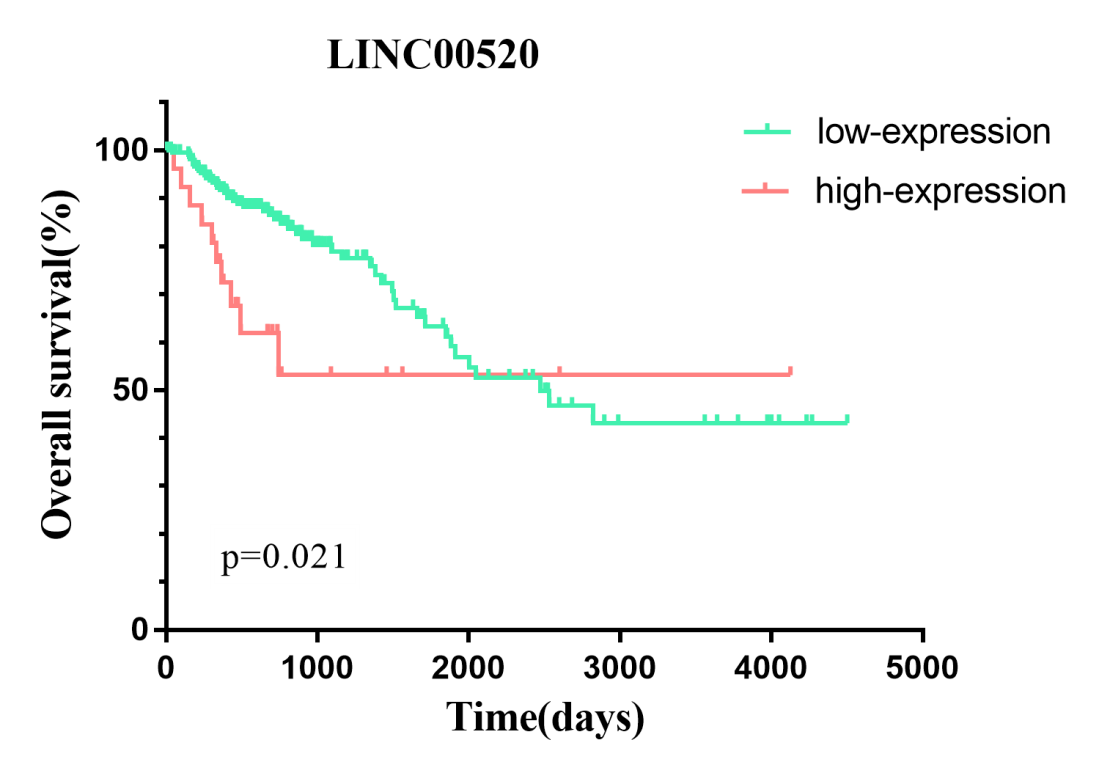


**Supplementary Fig S6** The survival curves of two groups of the colorectal cancer patients who highly and lowly expressed *LINC00520* gene, respectively.

**References**

Chen, B., Wang, C., Zhang, J., Zhou, Y., Hu, W., and Guo, T. (2018). New insights into long noncoding RNAs and pseudogenes in prognosis of renal cell carcinoma. *Cancer Cell Int* 18**,** 157. doi: 10.1186/s12935-018-0652-6.

Cross, D., Reding, D.J., Salzman, S.A., Zhang, K.Q., Catalona, W.J., Burke, J., et al. (2004). Expression and initial promoter characterization of PCAN1 in retinal tissue and prostate cell lines. *Med Oncol* 21(2)**,** 145-153. doi: 10.1385/mo:21:2:145.

Ding, X., Zhu, L., Ji, T., Zhang, X., Wang, F., Gan, S., et al. (2014). Long intergenic non-coding RNAs (LincRNAs) identified by RNA-seq in breast cancer. *PLoS One* 9(8)**,** e103270. doi: 10.1371/journal.pone.0103270.

Gomez-Maldonado, L., Tiana, M., Roche, O., Prado-Cabrero, A., Jensen, L., Fernandez-Barral, A., et al. (2015). EFNA3 long noncoding RNAs induced by hypoxia promote metastatic dissemination. *Oncogene* 34(20)**,** 2609-2620. doi: 10.1038/onc.2014.200.

Gong, J., Tian, J., Lou, J., Ke, J., Li, L., Li, J., et al. (2016). A functional polymorphism in lnc-LAMC2-1:1 confers risk of colorectal cancer by affecting miRNA binding. *Carcinogenesis* 37(5)**,** 443-451. doi: 10.1093/carcin/bgw024.

Gu, Y., Feng, C., Liu, T., Zhang, B., and Yang, L. (2018). The downregulation of lncRNA EMX2OS might independently predict shorter recurrence-free survival of classical papillary thyroid cancer. *PLoS One* 13(12)**,** e0209338. doi: 10.1371/journal.pone.0209338.

He, Y., Hu, H., Wang, Y., Yuan, H., Lu, Z., Wu, P., et al. (2018). ALKBH5 Inhibits Pancreatic Cancer Motility by Decreasing Long Non-Coding RNA KCNK15-AS1 Methylation. *Cell Physiol Biochem* 48(2)**,** 838-846. doi: 10.1159/000491915.

Hu, Y., Wang, J., Qian, J., Kong, X., Tang, J., Wang, Y., et al. (2014). Long noncoding RNA GAPLINC regulates CD44-dependent cell invasiveness and associates with poor prognosis of gastric cancer. *Cancer Res* 74(23)**,** 6890-6902. doi: 10.1158/0008-5472.Can-14-0686.

Huang, J., Xie, N., Huang, H., Yao, J., and Hu, W. (2018). Long noncoding RNA STXBP5-AS1 inhibits cell proliferation, migration, and invasion via preventing the PI3K/AKT against STXBP5 expression in non-small-cell lung carcinoma. *J Cell Biochem*. doi: 10.1002/jcb.28023.

Jiang, Y., Du, F., Chen, F., Qin, N., Jiang, Z., Zhou, J., et al. (2017). Potentially functional variants in lncRNAs are associated with breast cancer risk in a Chinese population. *Mol Carcinog* 56(9)**,** 2048-2057. doi: 10.1002/mc.22659.

Keshavarz, M., Asadi, M.H., and Riahi-Madvar, A. (2019). Upregulation of pluripotent long noncoding RNA ES3 in HER2-positive breast cancer. *J Cell Biochem* 120(10)**,** 18398-18405. doi: 10.1002/jcb.29152.

Liu, T., Zhang, X., Gao, S., Jing, F., Yang, Y., Du, L., et al. (2016). Exosomal long noncoding RNA CRNDE-h as a novel serum-based biomarker for diagnosis and prognosis of colorectal cancer. *Oncotarget* 7(51)**,** 85551-85563. doi: 10.18632/oncotarget.13465.

Liu, Y., Li, M., Yu, H., and Piao, H. (2019). LncRNA SRA1 is down-regulated in HPV-negative cervical squamous cell carcinoma and regulates cancer cell behaviors. *Biosci Rep* 39(8). doi: 10.1042/bsr20191226.

Luo, J., Xu, L.N., Zhang, S.J., Jiang, Y.G., Zhuo, D.X., Wu, L.H., et al. (2018a). Downregulation of LncRNA-RP11-317J10.2 promotes cell proliferation and invasion and predicts poor prognosis in colorectal cancer. *Scand J Gastroenterol* 53(1)**,** 38-45. doi: 10.1080/00365521.2017.1392597.

Luo, Y., Ouyang, J., Zhou, D., Zhong, S., Wen, M., Ou, W., et al. (2018b). Long Noncoding RNA GAPLINC Promotes Cells Migration and Invasion in Colorectal Cancer Cell by Regulating miR-34a/c-MET Signal Pathway. *Dig Dis Sci* 63(4)**,** 890-899. doi: 10.1007/s10620-018-4915-9.

Rose, A.M., Krishan, A., Chakarova, C.F., Moya, L., Chambers, S.K., Hollands, M., et al. (2018). MSR1 repeats modulate gene expression and affect risk of breast and prostate cancer. *Ann Oncol* 29(5)**,** 1292-1303. doi: 10.1093/annonc/mdy082.

Saha, S.K., Jeong, Y., Cho, S., and Cho, S.G. (2018). Systematic expression alteration analysis of master reprogramming factor OCT4 and its three pseudogenes in human cancer and their prognostic outcomes. *Sci Rep* 8(1)**,** 14806. doi: 10.1038/s41598-018-33094-7.

Shao, S., Wang, C., Wang, S., Zhang, H., and Zhang, Y. (2019). LncRNA STXBP5-AS1 suppressed cervical cancer progression via targeting miR-96-5p/PTEN axis. *Biomed Pharmacother* 117**,** 109082. doi: 10.1016/j.biopha.2019.109082.

Shi, D., Qu, Q., Chang, Q., Wang, Y., Gui, Y., and Dong, D. (2017). A five-long non-coding RNA signature to improve prognosis prediction of clear cell renal cell carcinoma. *Oncotarget* 8(35)**,** 58699-58708. doi: 10.18632/oncotarget.17506.

Shi, J., Li, X., Zhang, F., Zhang, C., Guan, Q., Cao, X., et al. (2015). Circulating lncRNAs associated with occurrence of colorectal cancer progression. *Am J Cancer Res* 5(7)**,** 2258-2265.

Shi, X., Tan, H., Le, X., Xian, H., Li, X., Huang, K., et al. (2018). An expression signature model to predict lung adenocarcinoma-specific survival. *Cancer Manag Res* 10**,** 3717-3732. doi: 10.2147/cmar.S159563.

Sun, R., Liu, Z., Tong, D., Yang, Y., Guo, B., Wang, X., et al. (2017). miR-491-5p, mediated by Foxi1, functions as a tumor suppressor by targeting Wnt3a/beta-catenin signaling in the development of gastric cancer. *Cell Death Dis* 8(3)**,** e2714. doi: 10.1038/cddis.2017.134.

Tracy, K.M., Tye, C.E., Ghule, P.N., Malaby, H.L.H., Stumpff, J., Stein, J.L., et al. (2018). Mitotically-Associated lncRNA (MANCR) Affects Genomic Stability and Cell Division in Aggressive Breast Cancer. *Mol Cancer Res* 16(4)**,** 587-598. doi: 10.1158/1541-7786.Mcr-17-0548.

Wang, F., Tian, X., Zhou, J., Wang, G., Yu, W., Li, Z., et al. (2018a). A threelncRNA signature for prognosis prediction of acute myeloid leukemia in patients. *Mol Med Rep* 18(2)**,** 1473-1484. doi: 10.3892/mmr.2018.9139.

Wang, M., Wang, M., Wang, Z., Yu, X., Song, Y., Wang, C., et al. (2018b). Long non-coding RNA-CTD-2108O9.1 represses breast cancer metastasis by influencing leukemia inhibitory factor receptor. *Cancer Sci* 109(6)**,** 1764-1774. doi: 10.1111/cas.13592.

Wang, X., Wang, L., Gong, Y., Liu, Z., Qin, Y., Chen, J., et al. (2019). Long noncoding RNA LINC01296 promotes cancer-cell proliferation and metastasis in urothelial carcinoma of the bladder. *Onco Targets Ther* 12**,** 75-85. doi: 10.2147/ott.S192809.

Wu, X., He, X., Li, S., Xu, X., Chen, X., and Zhu, H. (2016). Long Non-Coding RNA ucoo2kmd.1 Regulates CD44-Dependent Cell Growth by Competing for miR-211-3p in Colorectal Cancer. *PLoS One* 11(3)**,** e0151287. doi: 10.1371/journal.pone.0151287.

Wu, X., Wang, Y., Yu, T., Nie, E., Hu, Q., Wu, W., et al. (2017). Blocking MIR155HG/miR-155 axis inhibits mesenchymal transition in glioma. *Neuro Oncol* 19(9)**,** 1195-1205. doi: 10.1093/neuonc/nox017.

Xie, F., Xiang, X., Huang, Q., Ran, P., Yuan, Y., Li, Q., et al. (2017). Reciprocal control of lncRNA-BCAT1 and beta-catenin pathway reveals lncRNA-BCAT1 long non-coding RNA acts as a tumor suppressor in colorectal cancer. *Oncotarget* 8(14)**,** 23628-23637. doi: 10.18632/oncotarget.15466.

Xie, T., Pi, G., Yang, B., Ren, H., Yu, J., Ren, Q., et al. (2019). Long non-coding RNA 520 is a negative prognostic biomarker and exhibits pro-oncogenic function in nasopharyngeal carcinoma carcinogenesis through regulation of miR-26b-3p/USP39 axis. *Gene* 707**,** 44-52. doi: 10.1016/j.gene.2019.02.093.

Xu, G., Chen, J., Pan, Q., Huang, K., Pan, J., Zhang, W., et al. (2014). Long noncoding RNA expression profiles of lung adenocarcinoma ascertained by microarray analysis. *PLoS One* 9(8)**,** e104044. doi: 10.1371/journal.pone.0104044.

Xu, S., Wang, P., You, Z., Meng, H., Mu, G., Bai, X., et al. (2016). The long non-coding RNA EPB41L4A-AS2 inhibits tumor proliferation and is associated with favorable prognoses in breast cancer and other solid tumors. *Oncotarget* 7(15)**,** 20704-20717. doi: 10.18632/oncotarget.8007.

Xue, M., Shi, D., Xu, G., and Wang, W. (2019). The long noncoding RNA linc00858 promotes progress of lung cancer through miR-3182/MMP2 axis. *Artif Cells Nanomed Biotechnol* 47(1)**,** 2091-2097. doi: 10.1080/21691401.2019.1617728.

Zhong, B.L., Bian, L.J., Wang, G.M., Zhou, Y.F., Chen, Y.Y., and Peng, F. (2016). Identification of key genes involved in HER2-positive breast cancer. *Eur Rev Med Pharmacol Sci* 20(4)**,** 664-672.

Zuo, W., Zhang, W., Xu, F., Zhou, J., and Bai, W. (2019). Long non-coding RNA LINC00485 acts as a microRNA-195 sponge to regulate the chemotherapy sensitivity of lung adenocarcinoma cells to cisplatin by regulating CHEK1. *Cancer Cell Int* 19**,** 240. doi: 10.1186/s12935-019-0934-7.
